# Investigation of the stent induced deformation on hemodynamic of internal carotid aneurysms by computational fluid dynamics

**DOI:** 10.1038/s41598-023-34383-6

**Published:** 2023-05-02

**Authors:** Sajad Salavatidezfouli, As’ad Alizadeh, M. Barzegar Gerdroodbary, Amir Sabernaeemi, Amir Musa Abazari, Armin Sheidani

**Affiliations:** 1 Mathematics Area, MathLab, International School for Advanced Studies (SISSA), Trieste, Italy; 2 Department of Civil Engineering, College of Engineering, Cihan University-Erbil, Erbīl, Iraq; 3 Department of Mechanical Engineering, Babol Noshirvani University of Technology, Babol, Iran; 4 Department of Space, Earth and Environment, Chalmers University of Technology, Gothenburg, Sweden; 5 Department of Mechanical Engineering, Faculty of Engineering, Urmia University, Urmia, Iran

**Keywords:** Biomedical engineering, Mechanical engineering

## Abstract

Application of the stent for treatment of the internal carotid artery (ICA) aneurysms has been extensively increased in recent decades. In the present work, stent-induced deformations of the parent vessel of ICA aneurysms are fully investigated. This study tries to visualize blood stream and calculated hemodynamic factors inside the four ICA aneurysms after deformations of parent vessel. For the simulation of the non-Newtonian blood stream, computational fluid dynamic is applied with one-way Fluid–Solid interaction (FSI) approach. Four ICA aneurysms with different ostium sizes and neck vessel angle are selected for this investigation. Wall shear stress on wall of aneurysm is analyzed in two angles of deformation due to application of the stent. Blood flow investigation shows that the deformation of the aneurysm limited blood entrance to the sac region and this decreases the blood velocity and consequently oscillatory shear index (OSI) on the sac wall. It is also observed that the stent-induced deformation is more effective on those cases with extraordinary OSI values on aneurysm wall.

## Introduction

Usage of stent and coiling technique are the primary techniques for the treatment of the cerebral aneurysm since surgical clipping is high-risk approach for treatment specially patients with high ages. However, it is reported that recanalization may occur after endovascular embolization^1^. Late re-hemorrhage and a lower rate of thorough obliteration are two main challenges for usage of the endovascular coiling. These disadvantageous of endovascular coiling have been explained and resolved by new achievements^2,3^. The usage of stent is recognized as an important method for the reduction of the aneurysm rupture risk in patients with high sac section area. The main application of the stent usage is to avoid the main blood stream entering into aneurysm sac and consequently, the rupture risk of the aneurysm is reduced^4,5^.

Although several investigations have been done to compare these endovascular techniques, the selection of efficient method for treatment of various cases is still challenging^6,7^. The decisions of medical team are done based on some limited factors and their experience of is more prominent in final decision. As mentioned before, use of stent along with endovascular coiling could-significantly decrease the risk of rupture^8,9^. Stent not only preserve the coil fibre inside the aneurysm but also reformed parent vessel to reduce blood flow rate inside the sac section^10^. This function of the stent has motivated surgeons to apply this for most cases. However, details of deformation on the risk of aneurysm rupture were not investigated in full details^11,12^.

The concept of aneurysm deformation is highly important for the treatment of the patients^13^. The evaluation of the stent performance could be done via analysis of the main hemodynamic aspects of blood in the sac section area^14,15^. Jeong et al.^14^ investigated arbitrary secular geometry of aneurysm while real model of aneurysm has different geometrical features. Ullery et al.^15^ quantified the geometry and respiration-induced deformation of abdominal branch vessels and stents after fenestrated (F-) and snorkel (Sn-) endovascular aneurysm repair. However, hemodynamic analyses is required for investigated deformations. Sabernaeemi et al.^16^ and Voss et al.^17^ investigated hemodynamic in real 3-D models but limited computational results are presented about angle of deformation on the hemodynamic of blood stream.

In this study, the computational fluid dynamic is employed for the visualization of the blood flow in four different ICA aneurysms. Blood pulsatile flow is considered for the simulation of the blood stream in the real geometry of the selected ICA aneurysms. Two deformation stages are chosen as the post-interventional models for aneurysms after usage of stent. Wall shear stress and OSI index are compared and analyzed.

## Governing equation and computational technique

It is confirming that all methods were carried out in accordance with relevant guidelines and regulations. Besides, all experimental protocols were approved by of the Ca' Granda Niguarda Hospital and it is confirmed that informed consent was obtained from all subjects and/or their legal guardian(s). This study selected geometry of aneurysm from Aneurisk website^18^.

**Figure 1:**
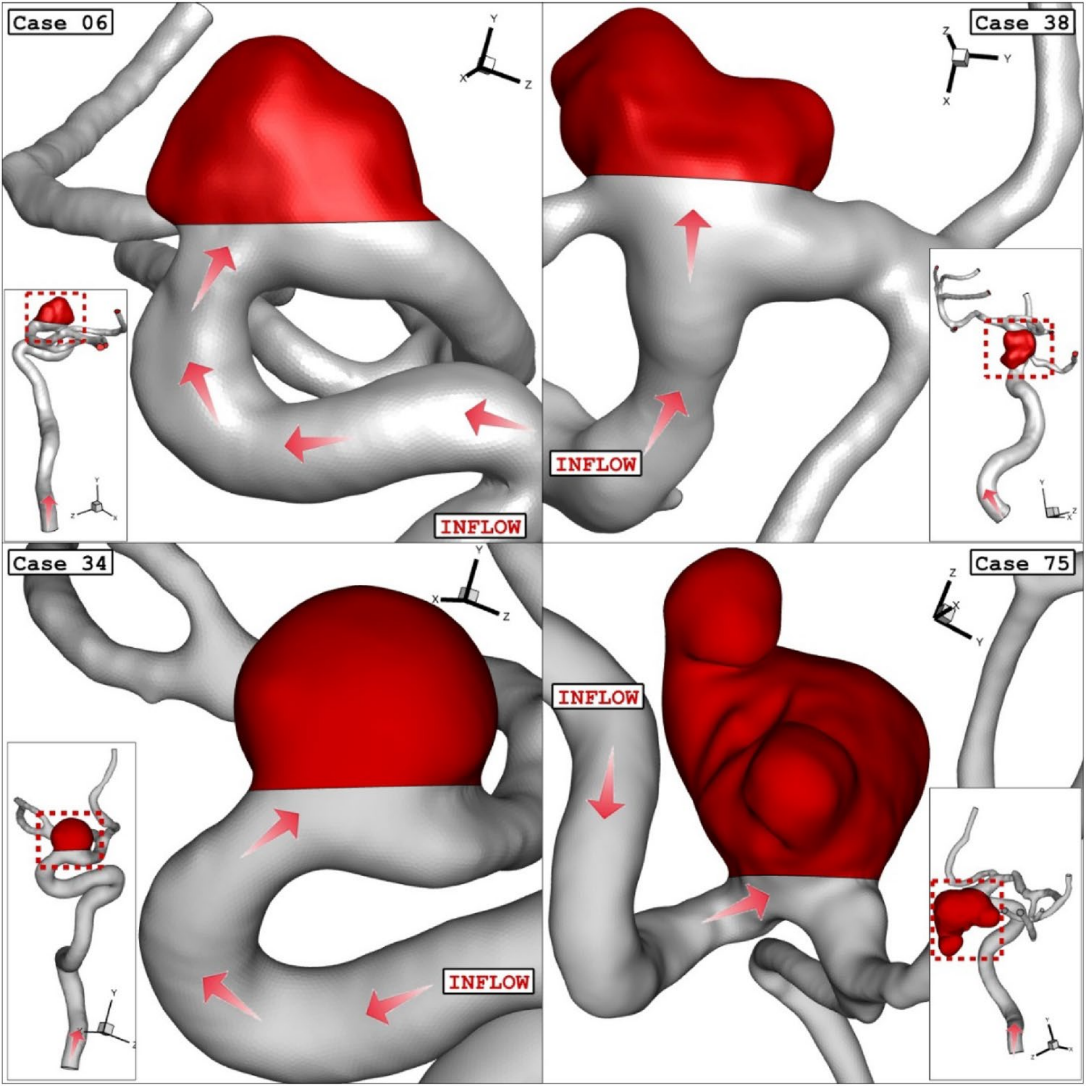
ICA aneurysm geometry of 4 different cases (main models: before deformation).

This research study has focused on the different stages of deformation by hemodynamic analysis of blood stream. Computational fluid dynamic is used for the investigation of the blood hemodynamic of four distinctive cases as illustrated in Fig. 1.

**Figure 2:**
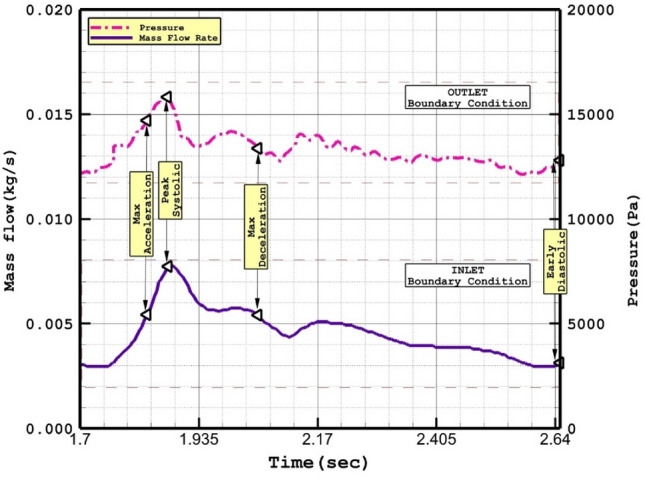
Applied mass and pressure profile at inlet and outlets^29^.

Computational and theoretical techniques are extensively used in various biology science and biomedical systems^19–28^ Simulation of the transient blood stream inside the chosen ICA aneurysms is done via simple algorithm in ANSYS-FLUENT software. The interaction of the blood and vessel is model via One-way FSI in which the blood force influences on the aneurysm wall as an exterior force. One-Way FSI implies the effect of the fluid on to the solid and the solid deforms. In fact, pressure of blood near wall is considered as an exterior force in the solid part and it would results in deformation. Owing to pulsatile feature of blood flow inside the vessels, mass flow rate at inlet and pressure value at outlet is applied by displayed pattern in Fig. 2. Casson model is used for modeling of the blood viscosity^29^. We used a correlation for calculation of the viscosity (Casson model) in relation with Hematocrit value as follow:

1 $$\mu =0.1\left({\left[\sqrt{\eta }+\sqrt{{\tau }_{\gamma }\left(\frac{1-{e}^{-m\left|\dot{\gamma }\right|}}{\left|\dot{\gamma }\right|}\right)}\right]}^{2}\right)and\; {\tau }_{\gamma }={\left(0.625H\right)}^{3}$$

In this model, effect of Hematocrit (H) is also applied for the estimation of the viscosity^30,31^.

**Figure 3:**
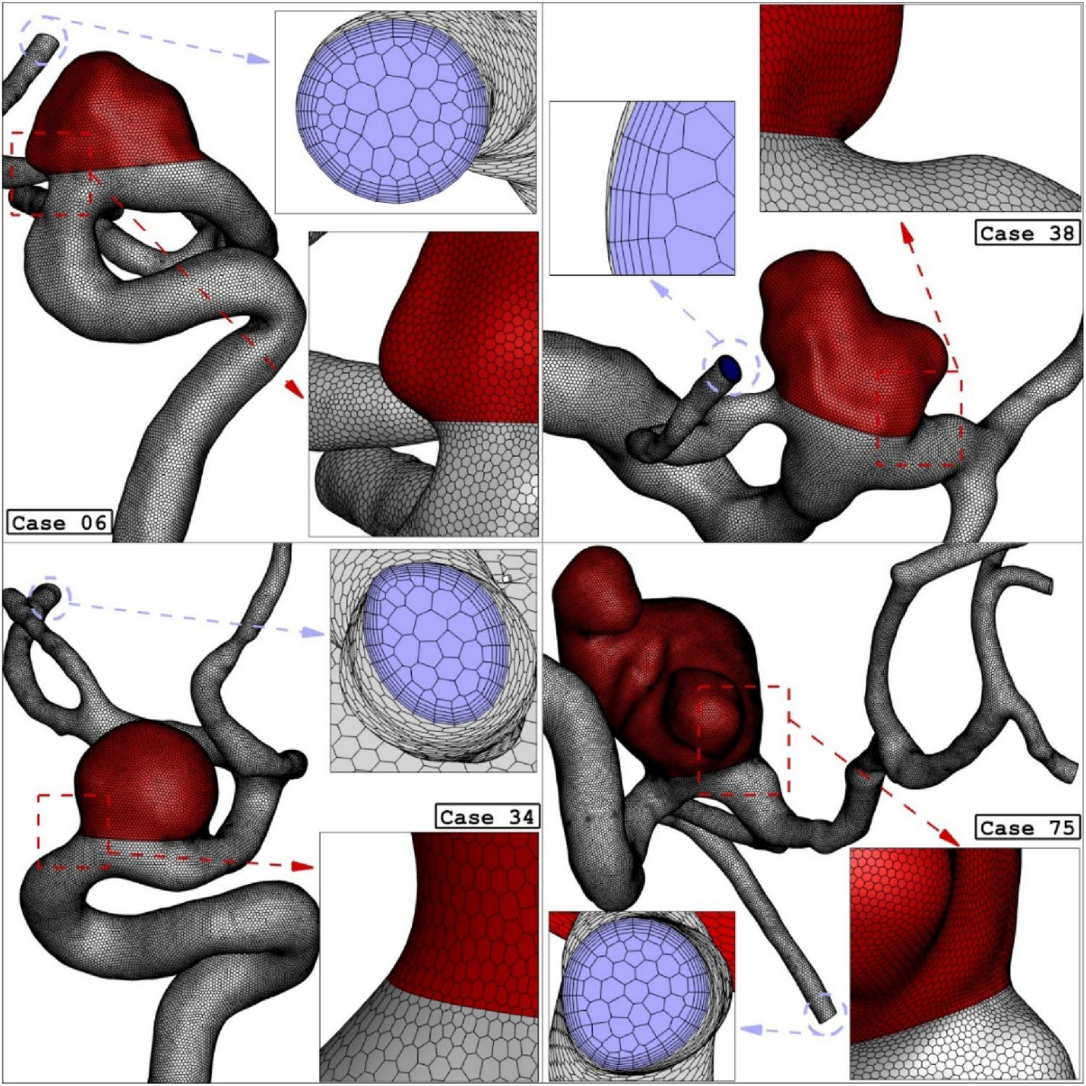
Grid generation for 4 different ICA cases (main models: before deformation).

Figure 3 displayed the produced grid for the selected ICA aneurysms. Boundary layer is applied for the grid production near the aneurysm wall^32–35^. The resolution of applied grid near the wall inside the aneurysms is higher than other sections due to its important on the archived results^36–40^. The close-up view of the applied grid is also displayed to demonstrate the resolution of the grids^41–44^. The grid study is also performed to ensure about the grid independency. Table 1 presents results of grid study for four cases by comparing the change of average WSS on sac wall.

**Table 1:** Grid study.

Case	Element size (mm)	Number of elements	Run-time (h)	Ave WSS on Sac (Pa)	Change %
Case 06	0.24	85,476	2	6.04	–
	0.20	165,478	4	8.02	32.8
	0.16	298,201	6	9.13	13.8
	0.12	654,781	14	9.22	1.0
Case 38	0.22	121,459	2	5.17	–
	0.18	226,987	4	7.34	42.0
	0.14	402,161	8	8.47	15.4
	0.10	805,794	18	8.51	0.5
Case 34	0.24	96,234	2	6.34	–
	0.20	181,210	4	8.42	32
	0.16	281,320	6	9.04	7
	0.12	632,180	14	9.62	6
Case 75	0.22	122,220	2	5.94	–
	0.18	220,420	4	8.22	38
	0.14	440,204	6	9.43	14
	0.10	820,410	14	9.82	5

The ICA aneurysms are sorted by the ostium section area and all selected cases are related to female patients in which blood hematocrit value is 0.4 as presented in Table 2. In this table, age, neck vessel angle and ostium section area of the chosen ICA geometries are presented. The main concept for the selection of these aneurysms are neck vessel angle and ostium section area that are presented in Table 2. The range neck vessel angle is within 26–45 degree, while ostium section area varies between 35 and 53.2 mm^2^ in the chosen models. The presented results of OSI value are calculated at the end of 3rd blood cycles while the pressure, WSS and average blood velocity are reported on the peak systolic stage on the 3rd cycle where the blood stream is maximum^45,46^. Since these four hemodynamic factors (i.e. OSI, WSS, pressure and velocity) play critical role on the hemodynamic of blood stream and they could use to examine the risk of aneurysm rupture, they are chosen in our investigations^47,48^.

**Table 2:** Selected aneurysm cases.

Case ID	Ostium section area (mm^2^)	Neck vessel angle (degree)	Sex	Age (years)
06	34.9	31.4	Female (HCT = 0.40)	45
38	38.4	45.4	Female (HCT = 0.40)	74
34	41.3	39.8	Female (HCT = 0.40)	42
75	53.2	26.6	Female (HCT = 0.40)	74

**Figure 4:**
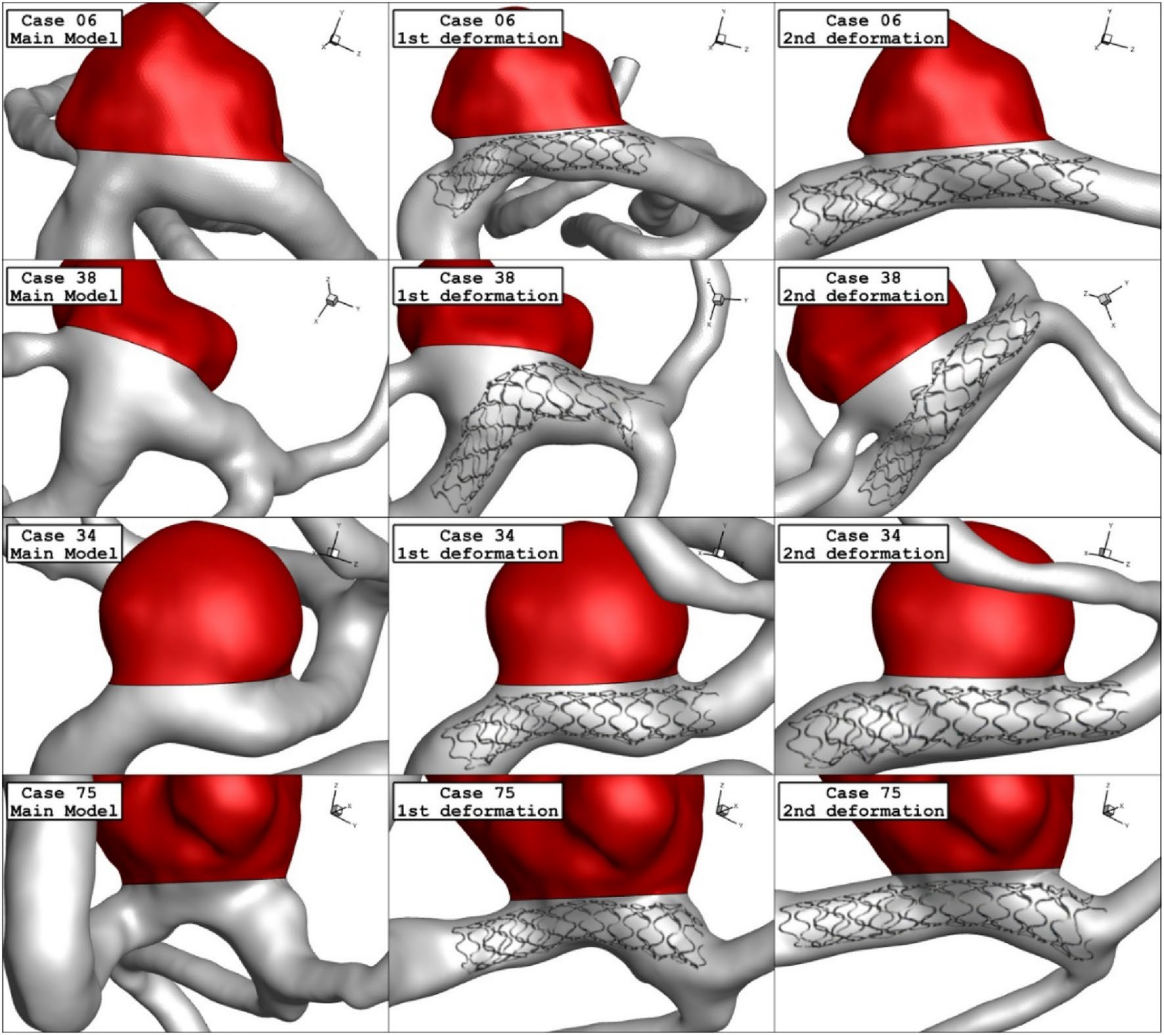
Geometry of ICA models and their deformations.

**Figure 5:**
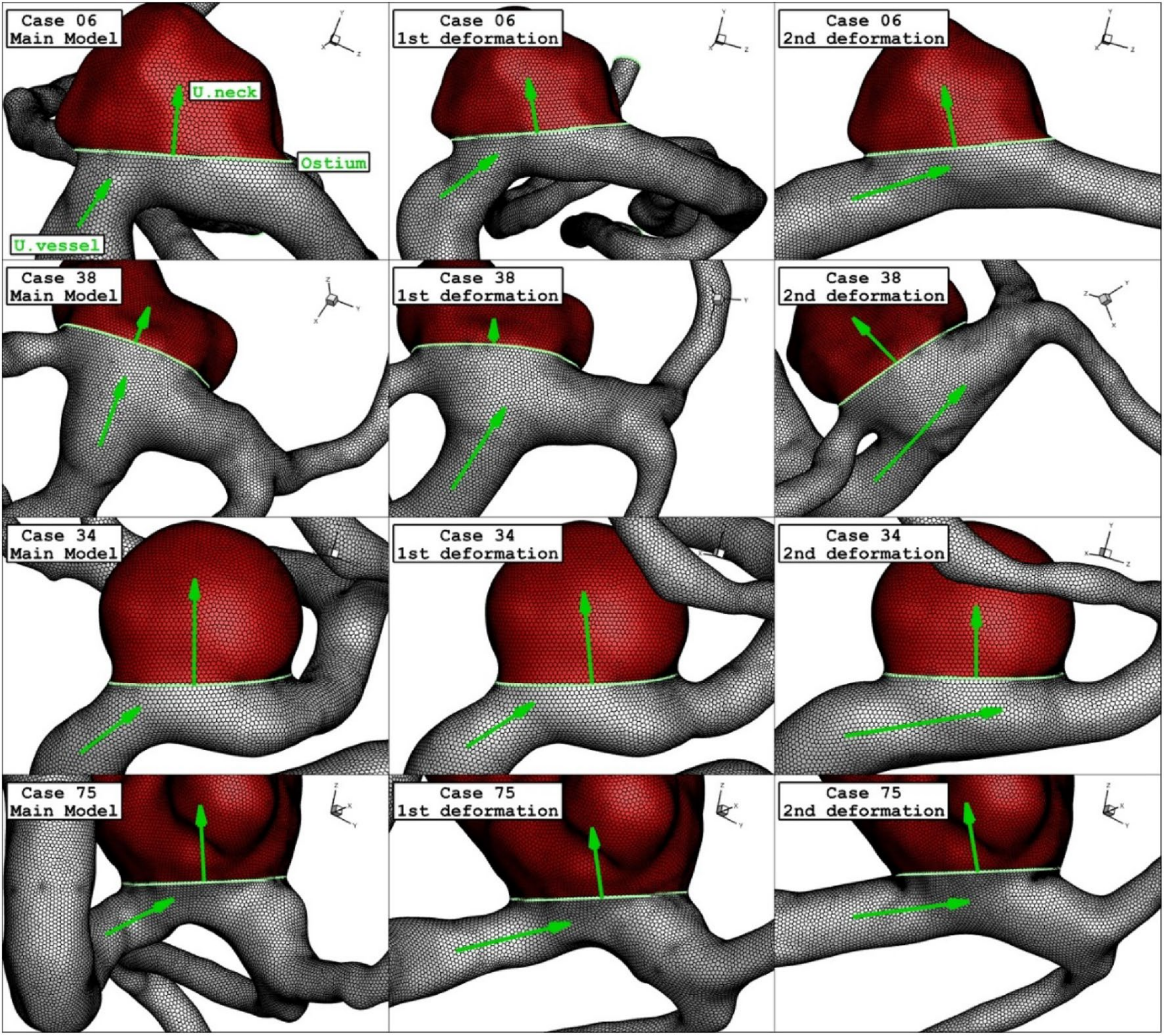
Grid generation of ICA models and their deformations.

The deformed aneurysms are displayed in Fig. 4 for the chosen ICA aneurysms. Two post-interventional models are created in which the usage of the stent aligned the parent vessel and reduce the neck vessel angle. As demonstrated in Fig. 5, the angle of parent vessel with ostium plane is reduced in the post-interventional models. Details of deformed aneurysms are presented in Table 3.

**Table 3:** Results of main parameters after and before deformation.

	Model number	Deformation	WSS (Pa)	OSI	Mean wall pressure (Pa)	Mean aneurysm Velocity (m/s)
Case06	1	Main case	24.5	0.026	23,760	0.76
	2	1st deformation	22.3	0.02	23,605	0.71
	3	2nd deformation	6.7	0.031	23,893	0.26
Case38	1	Main case	5.79	0.075	19,269	0.26
	2	1st deformation	4.69	0.077	20,005	0.21
	3	2nd deformation	3.77	0.030	20,118	0.16
Case34	1	Main case	21.12	0.002	25,456	0.75
	2	1st deformation	18.40	0.004	25,542	0.69
	3	2nd deformation	7.86	0.016	26,281	0.39
Case75	1	Main case	8.19	0.023	22,350	0.32
	2	1st deformation	6.28	0.023	22,313	0.22
	3	2nd deformation	2.25	0.021	22,644	0.10

## Results and discussion

In present work, the hemodynamic of the blood stream inside the original aneurysm and two deformed ones is investigated by comparison of the WSS, pressure and average blood velocity. The results of the mean WSS, OSI, mean wall pressure and mean velocity inside the aneurysms are presented in Table 2. In the following, figures are presented to illustrate and compare the achieved results for different models at various deforming stages.

**Figure 6:**
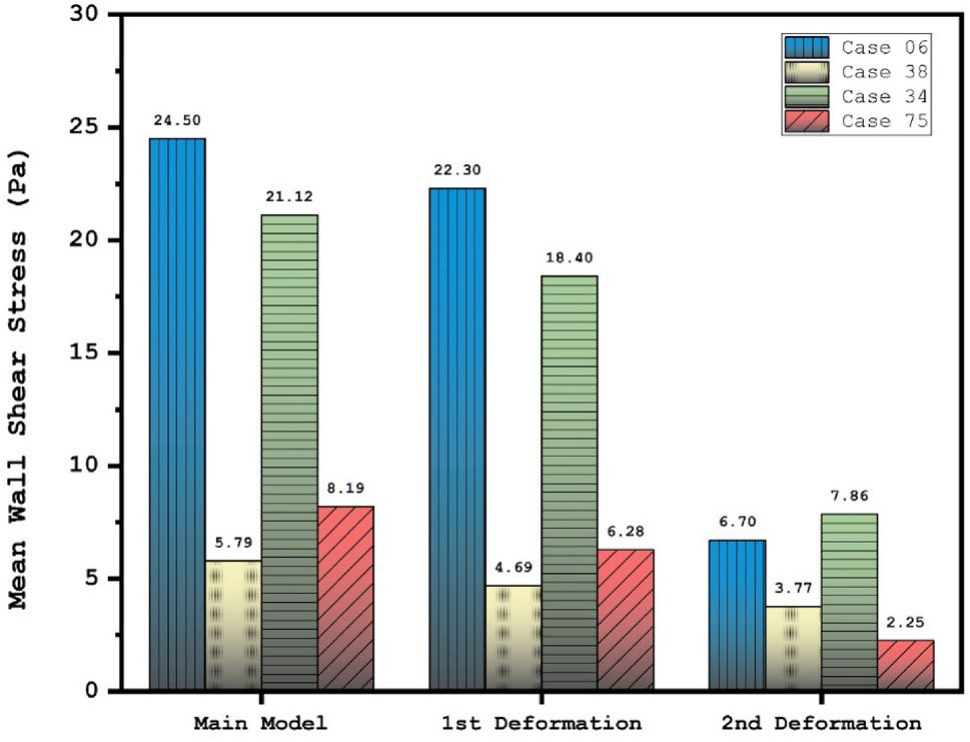
Deformation effects on mean values of wall shear stress (WSS) at peak systolic.

**Figure 7:**
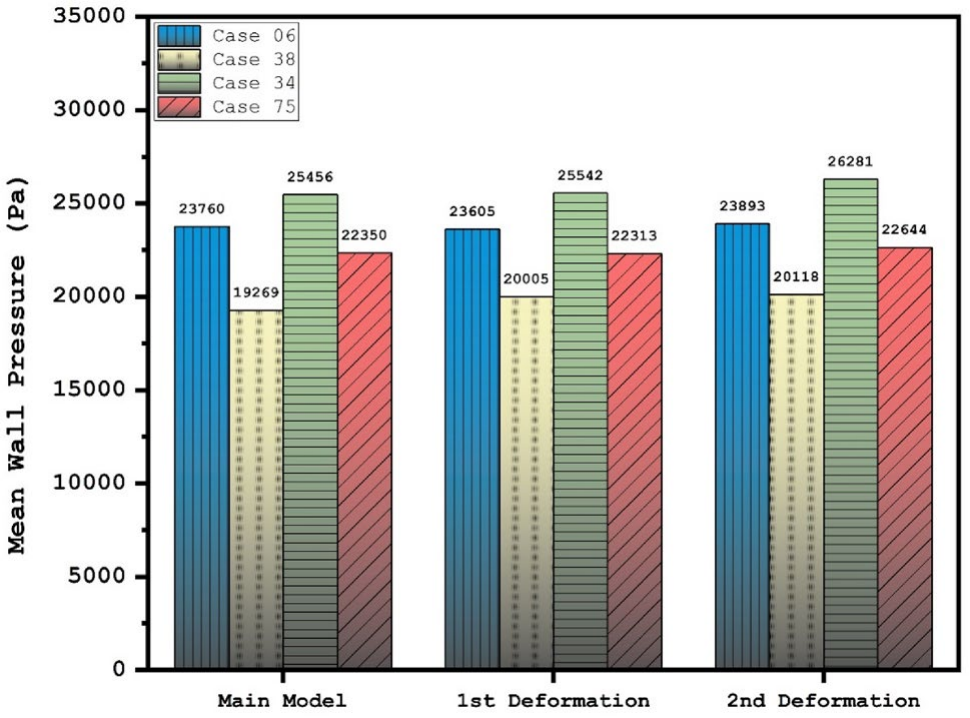
Deformation effects on mean values of sac wall pressure (Pa) at peak systolic.

Figure 6 and 7 illustrates the effects of 1st and 2nd deformation on the mean wall shear stress and sac wall pressure of selected ICA cases at peak systolic, respectively. Although the ostium size is not similar in the chosen aneurysms, it is found that the impacts of the 1st deformation on mean WSS is limited. After second deformation, the value of mean WSS substantially reduces on sac wall while average pressure on the aneurysm wall (Fig. 7) is not changed noticeable. In two cases of 06 and 34, the effects of 2nd deformation results in more than 65% WSS reduction.

**Figure 8:**
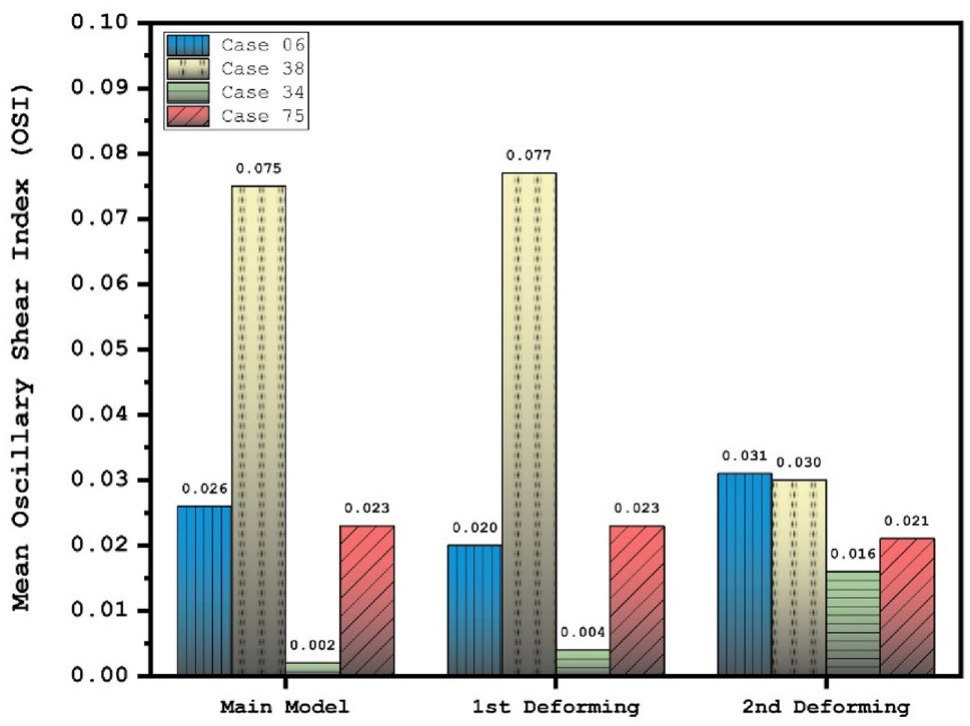
Deformation effects on mean values of OSI at early diastolic.

**Figure 9:**
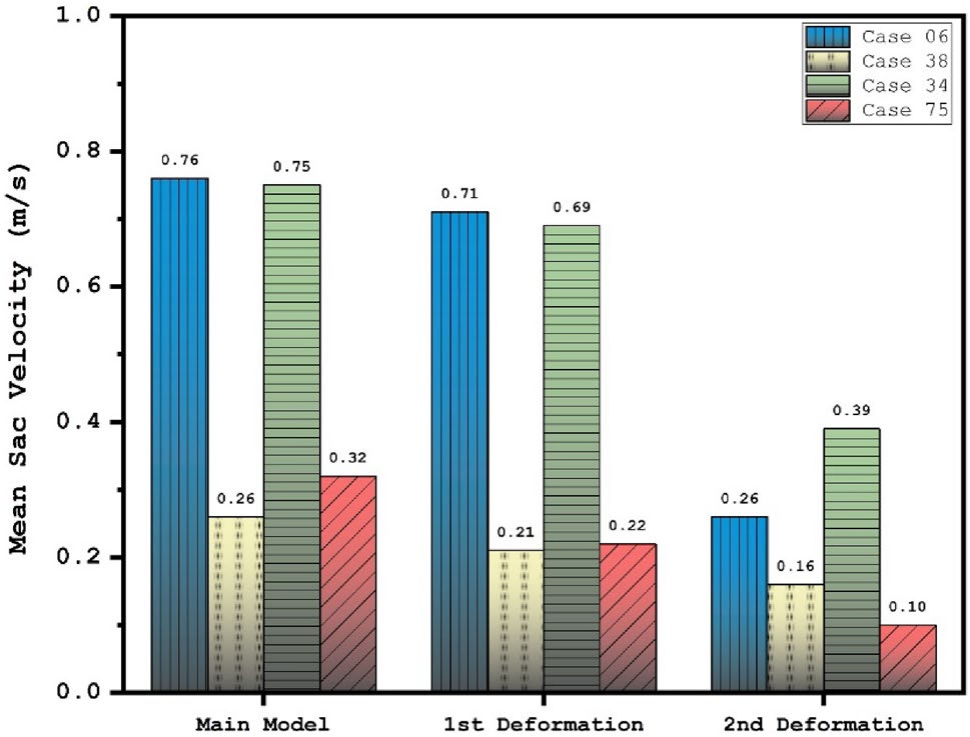
Deformation effects on mean values of sac velocity at peak systolic.

Effects of 1st and 2nd deformation on mean OSI (early diastolic) and sac velocity (peak systolic) are presented in Figs. 8 and 9. Obtained results indicates that the OSI index decreases after 2nd deformation for cases with extremely high values (case 38) while other cases has limited effects. This finding confirms that usage of stent as technique for the deformation of the parent vessel is effective for decrease the risk of aneurysm rupture. Results of mean sac velocity also verify that the stent deformation is effective on those cases with high velocity values.

**Figure 10:**
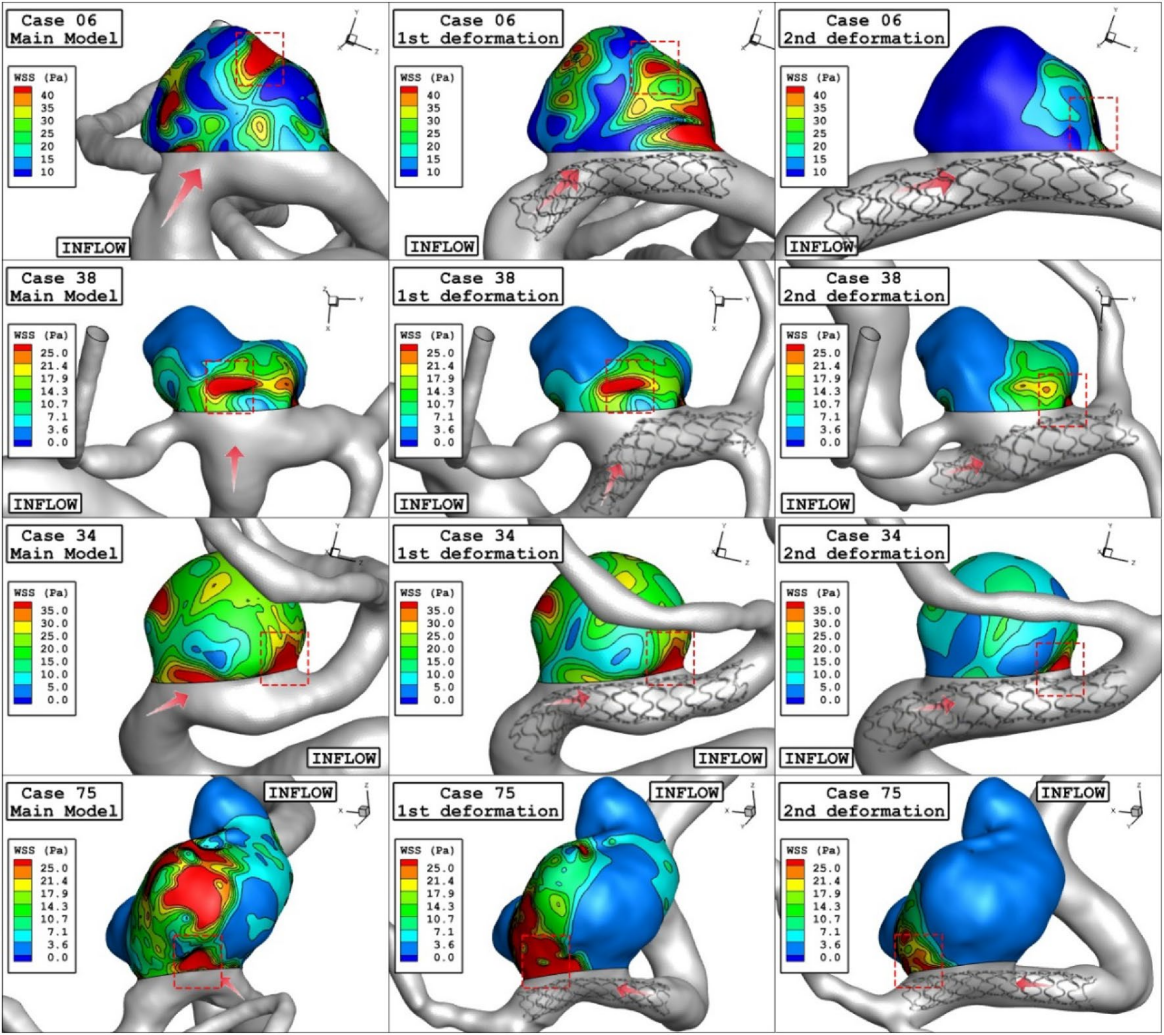
WSS contours (Peak systolic) in different neck vessel angle.

**Figure 11:**
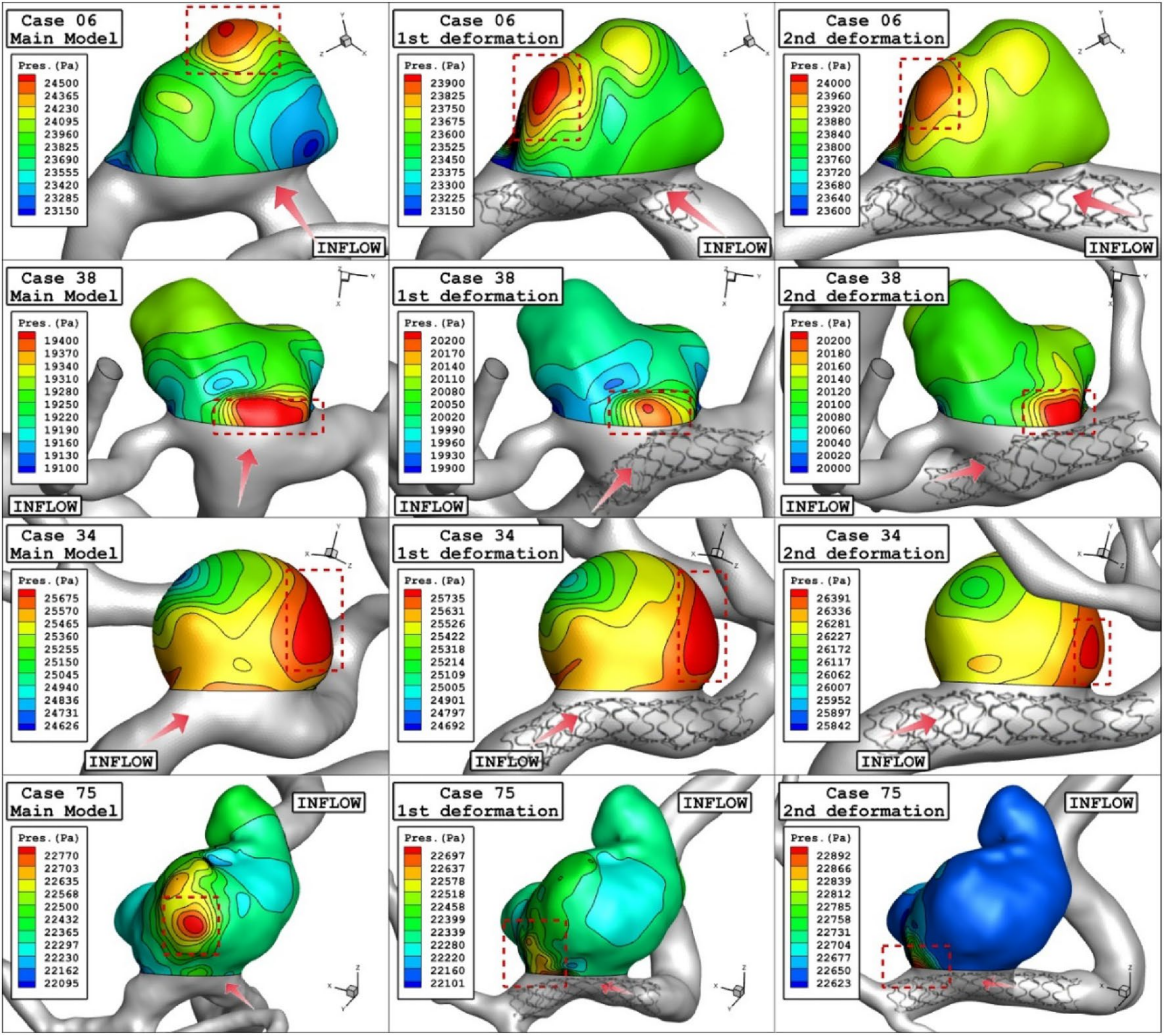
Wall pressure contours (Peak systolic) in different neck vessel angle.

Figure 10 illustrates the distribution of WSS on sac wall for four cases after 1st and second deformations. The value and size of WSS in applied stent model clear are decreased due to lower blood flow rate inside the aneurysm. The contour also shows that the critical region after deforming is limited to the region near ostium. Comparison of the pressure distribution on the sac surface (Fig. 11) also shows that the average pressure value on the sac surface does not change substantially after post-interventional although blood entrance is limited owing to deformation.

**Figure 12:**
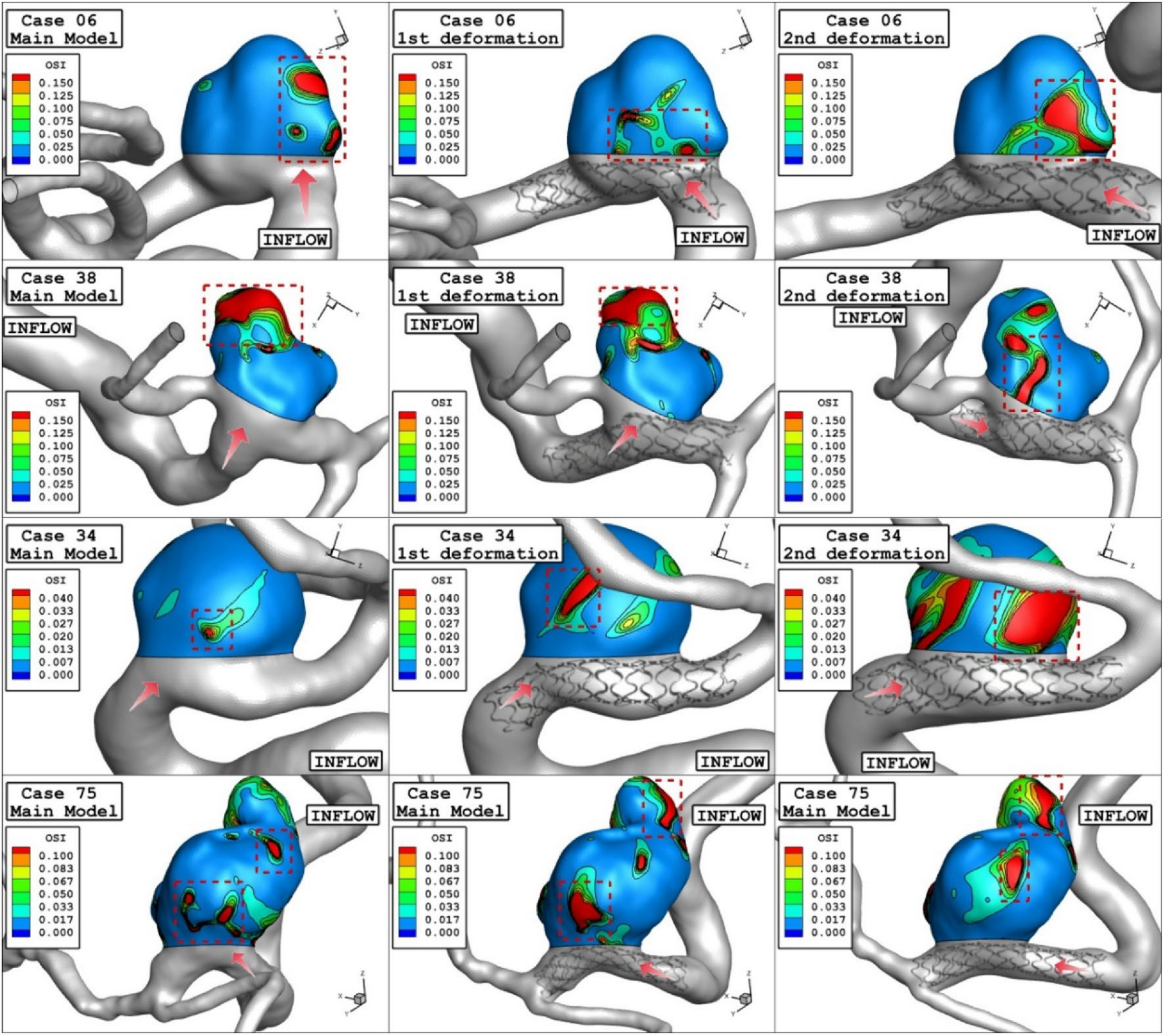
OSI contours (Early diastolic) in different neck vessel angle.

**Figure 13:**
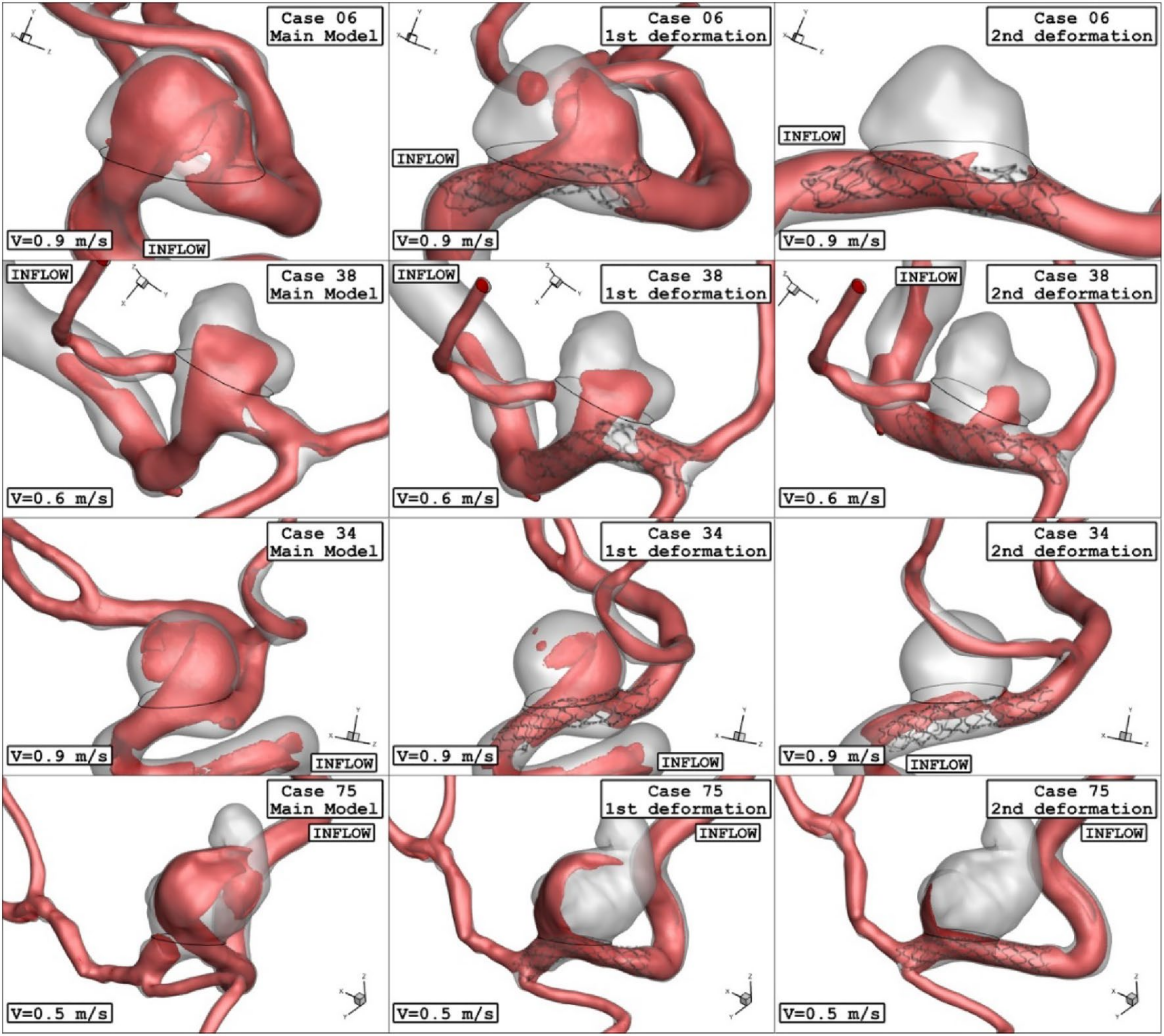
Iso-Surface (velocity at peak systolic) in different neck vessel angle.

Figure 12 depicts the variation of the OSI on sac surface at early diastolic in the selected ICA aneurysms. Obtained results show that the high OSI region may be extended after deformation in some cases. This effects may be related to low velocity of blood stream inside the aneurysm after deformation. The effects of stent-induced deformation on the blood structure are presented in Fig. 13 which demonstrates the blood structure by iso-velocity surface. Comparison of blood structure represents that significant reduction in the blood velocity inside the aneurysm. Indeed, the usage of stent which deformed the parent vessel would block blood entrance to sac section area and this is helpful for-reduction of the rupture.

**Figure 14:**
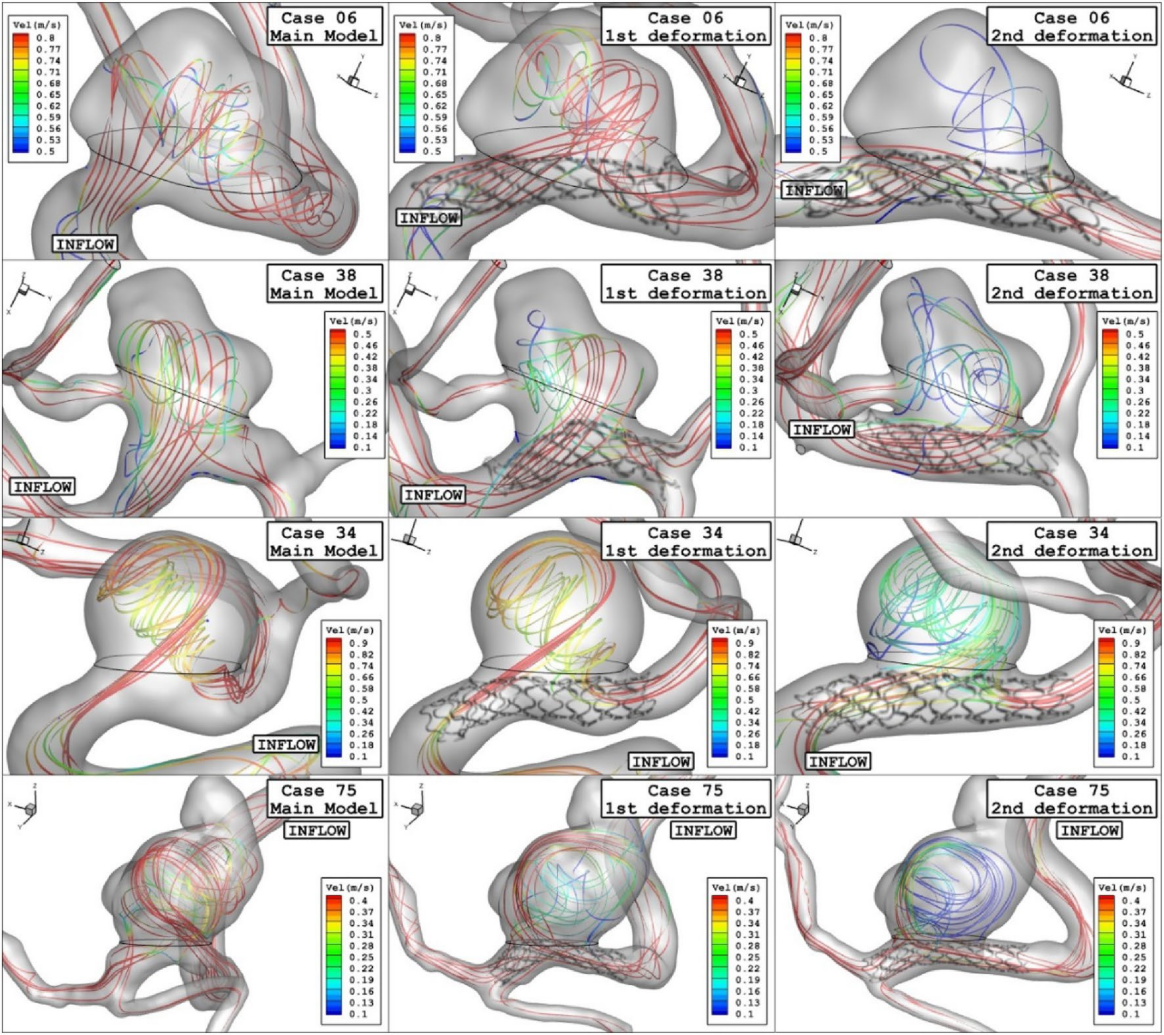
Streamlines (velocity at peak systolic) in different neck vessel angle.

Figure 14 demonstrates the blood stream inside the sac and it is colored by the velocity value. Comparison of the streamline confirm substantial velocity reduction of blood stream inside the sac after 2nd deformation in all cases.

## Conclusion

The aim of this study is to analyze the impacts of stent-induced deformation on the hemodynamic of the blood stream inside the sac section of four different ICA aneurysm. Comprehensive hemodynamic investigations are done to reveal the main changes in the blood stream after two stages of deformations. Selected sacs are chosen based on different ostium sizes and neck vessel angles. Computational fluid dynamic is applied for the hemodynamic study of the blood within the ICA aneurysms. OSI, WSS and pressure variations on the sac surface are displayed and investigated. The main advantageous of stent-deformation is formation of clot which prevent the entrance of blood flow. In addition, blood flow streams are compared for selected models of ICA aneurysms in two steps of deformations. Achieved results indicates that stent-induced deformation of the ICA cases significantly decreases OSI on aneurysms with extremely high OSI values. Besides, the velocity of the blood is reduced substantially when aneurysm deformation happens.

## Data Availability

Data are available upon reasonable request. Any additional data regarding this submission can be requested from the corresponding author via email.
