# Author Correction: Investigation of the stent induced deformation on hemodynamic of internal carotid aneurysms by computational fluid dynamics

**DOI:** 10.1038/s41598-023-35240-2

**Published:** 2023-05-17

**Authors:** Sajad Salavatidezfouli, As’ad Alizadeh, M. Barzegar Gerdroodbary, Amir Sabernaeemi, Amir Musa Abazari, Armin Sheidani

**Affiliations:** 1 Mathematics Area, MathLab, International School for Advanced Studies (SISSA), Trieste, Italy; 2 Department of Civil Engineering, College of Engineering, Cihan University-Erbil, Erbīl, Iraq; 3 Department of Mechanical Engineering, Babol Noshirvani University of Technology, Babol, Iran; 4 Department of Space, Earth and Environment, Chalmers University of Technology, Gothenburg, Sweden; 5 Department of Mechanical Engineering, Faculty of Engineering, Urmia University, Urmia, Iran

Correction to: *Scientific Reports* https://doi.org/10.1038/s41598-023-34383-6, published online 02 May 2023

The original version of this Article contained a spelling error in the name of the author As’ad Alizadeh which was incorrectly given as Asad Alizadeh.

The original Article has been corrected.

